# A case of synchronous bilateral breast cancer with different pathological responses to neoadjuvant chemotherapy with different biological character

**DOI:** 10.1186/2193-1801-2-272

**Published:** 2013-06-21

**Authors:** Mitsuhiro Hayashi, Yutaka Yamamoto, Noboru Takata, Hirotaka Iwase

**Affiliations:** Department of Breast and Endocrine Surgery, Kumamoto University Graduate School of Medical Sciences, 1-1-1 Honjo, Kumamoto-city, Kumamoto, 860-8556 Japan; Department of Surgery, Amakusa Medical Center, 854-1 Syokuba Kamebamachi, Amakusa-city, Kumamoto, 863-0046 Japan

**Keywords:** Neoadjuvant therapy, Estrogen receptor, HER2, Bilateral breast cancer

## Abstract

We report a case of synchronous locally advanced bilateral breast cancer with different pathological responses to neoadjuvant chemotherapy with different biological character. The patient had presented bilateral breast cancer: the left breast cancer was hormone receptor negative, human epidermal growth factor receptor-2 (HER2) positive, and classified as T4bN1M0, stage IIIb, while the right was hormone receptor positive, HER2-negative, and classified as T4bN0M0, stage IIIb. We administered four cycles of anthracycline-based therapy followed by 12 weekly cycles of taxane with trastuzumab for neoadjuvant chemotherapy. We had achieved a significant left tumor reduction after each chemotherapy, but not right tumor. Bilateral modified radical mastectomies with axillary lymph-node dissection were performed. The therapeutic effect in the left was determined as a pathological complete response, in contrast to the right side. She has no recurrence for more than five years, though she had advanced cancer with oncologic emergency. This case could be an informative experience to understand the relation of tumor biology and response to systemic therapy.

## Introduction

The escalation of targeting therapy in breast cancer is supported by a research of tumor biology. To ensure breast cancer patients receive optimal treatment, the elucidation of biology from variety fields, such as experimental and clinical research, has been advancing around world. Estrogen receptor (ER), Progesterone receptor (PgR), and human epidermal growth factor receptor-2 (HER2) are establish as strong predictive factors to induct each molecular targeting therapy, such as endocrine therapy and anti-HER2 therapy (Iwase 2008) (Hudis 2007). Moreover, these biological markers have also been investigated to be concerned with the effects of cytotoxic chemotherapy.

The number of enormous clinical trials have been designed to explore optimal treatment and to resolve the relation of tumor biology with systemic therapy, recently, neoadjuvant chemotherapy (NAC) attracts a high level of interest instead those of metastatic or adjuvant setting. NAC is considered to be the most practical an in vivo measure of chemo-sensitivity and could be procedure to evaluate relation of biology with treatment response, meanwhile NAC reduces tumor size and enables breast cancer patients to select breast conserving surgery (Mauri et al. 2005).

The clinical research of NAC is mostly based on clinical trials which consist of a variety of patient groups, and larger patient samples of well annotated are demanded to exclude each individual host characteristics. In this time, we experienced a valuable treatment case, which could reveal a correlation between tumor biology and response to NAC without host characteristic. The patient had presented synchronous locally advanced bilateral breast cancers with different biological markers, and achieved different tumor reduction effects. The therapeutic background of each breast cancer were similar condition except for the tumor characteristics, therefore, we reported this meaningful or educational case to understand the relation of tumor biology and sensitivity of NAC. Furthermore we also discussed the relation of NAC response with patient’s prognosis because it is still under controversial.

## Case report

**Figure 1:**
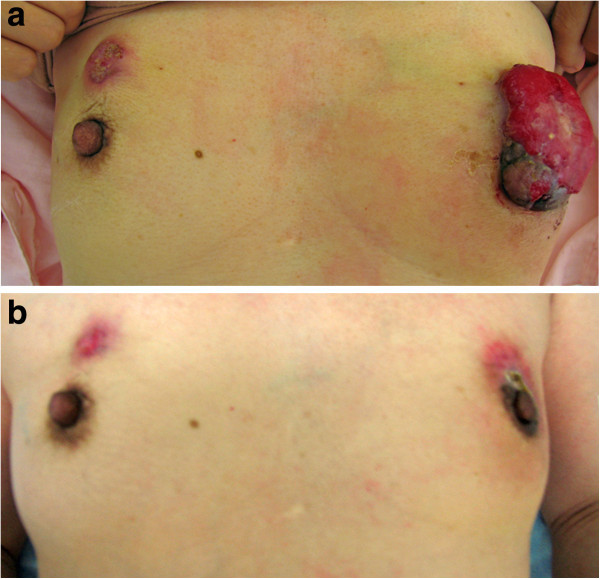
**Appearance of the bilateral breast cancers at pre-and post-treatment.** The right breast cancer was defined HR-positive and HER2-negative status at pre-treatment (**a**). After neoadjuvant chemotherapy, the right breast cancer presented no significant change (**b**). The left breast cancer was defined HR-negative and HER2-positive at pre-treatment (**a**). After neoadjuvant chemotherapy with trastuzumab, the left breast cancer revealed significant reduction (**b**).

**Figure 2:**
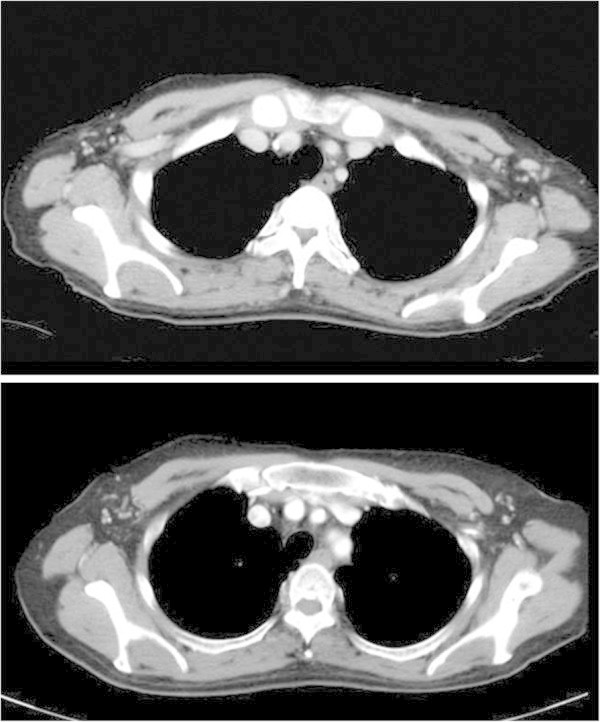
**CT findings of the bilateral axillary lymph nodes at pre-and post-treatment.** At the start of treatment, several lymph nodes in the left axilla were clearly swelling indicating metastasis (upper). After neoadjuvant chemotherapy those were not swelling (lower).

**Figure 3:**
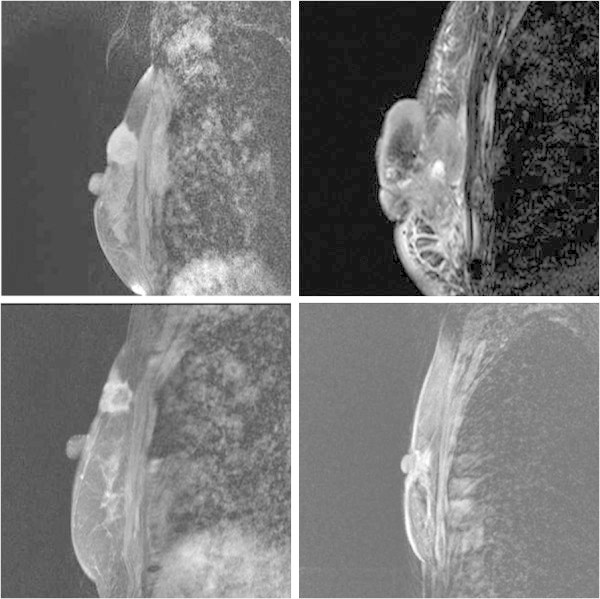
**MRI findings of the bilateral breast cancers at pre-and post-treatment.** MRI showed a distinctly round contrasting mass of 27 × 19 mm in the right breast (upper right) and a distinctly irregular contrasting mass of 57 × 42 mm in the left breast (upper left) at pre-treatment. Each mass had clearly skin invasion, by contrast each had not reached to the chest wall. After the treatment the left tumor showed a significant tumor reduction (lower left), but not right tumor (lower right).

A 60-year-old woman presented with locally advanced bilateral breast masses that she had noticed in her left breast 14 months earlier and in her right breast 2 months earlier. The left mass was 70 × 60 mm in diameter with blood oozing from skin exposed lesion, and the right mass was 34 × 32 mm in diameter with skin invasion (Figure 1a). She was seen in the first clinic because of oncologic emergency with severe anemia (hemoglobin 3.3 mg/ dl), atrial fibrillation and heart failure. The diagnosis of breast cancers were made from core needle biopsy, and the items investigated were histological type, nuclear grade, ER (6 F11, Ventana), PgR (16, Ventana), and HER2 overexpression (CB11, Ventana). A computed tomography scan revealed no distant metastasis, although several lymph nodes in the left axilla were clearly swelling indicating metastasis (Figure 2). MRI showed a distinctly irregular contrasting mass of 57 × 42 mm in the left breast, and a distinctly round contrasting mass of 27 × 19 mm in the right breast, nevertheless each mass had not reached to the chest wall (Figure 3). Serum tumor marker CA15-3 was slightly elevated 37.7 U/ ml (upper limit of normal 25.0 U/ mL), but not CEA and N-ST439. She had no family history of breast and ovarian cancer.

The pretreatment diagnosis were synchronous locally advanced bilateral breast cancers: the left breast cancer was defined invasive ductal carcinoma, ER-negative, PgR-negative, and HER2-positive classified as T4bN1M0, stage IIIb, while the right breast cancer was defined invasive ductal carcinoma, ER-positive, PgR-positive, and HER2-negative classified as T4bN0M0, stage IIIb (Table 1).

**Table 1:** **Tumor characteristics of the bilateral breast cancer**

	Right tumor		Left tumor	
Characteristics	Pre-NAC	Post-NAC	Pre-NAC	Post-NAC
Tumor Size	34 mm	25 mm	70 mm	0 mm
ER positivity	80%	90%	0%	-
PgR positivity	40%	60%	0%	-
HER2	1+	2+ FISH 1.2 fold	3+	-
Nuclear Grade	2	1	3	-

*NAC* neoadjuvant chemotherapy, *ER* estrogen receptor, *PgR* progesterone receptor.

We administered four cycles of fluorouracil (500 mg/m^2^), epirubicin (100 mg/m^2^) and cyclophosphamide (500 mg/m^2^) every three weeks (FEC), followed by concurrent weekly trastuzumab (4 mg/kg on day 1 and subsequent infusions at a dose of 2 mg/kg) with 12 weekly cycles of paclitaxel (80 mg/m^2^) for neoadjuvant chemotherapy (wPac + T). Informed consent was obtained from the patient before treatment.

**Figure 4Figure 4:**
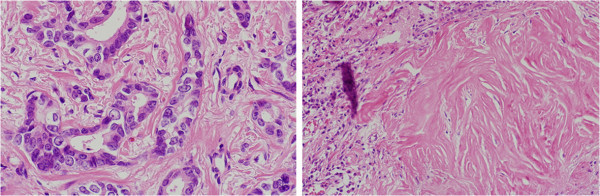
**Pathological findings of the bilateral breast cancers after treatment.** In the right surgical specimen, there were residual invasive cancer cells with no significant changes (right). In contrast, no cancer cells were observed in the left surgical specimen (left).

We had achieved a significant left tumor reduction after each chemotherapy, but not right tumor (Figure 1b). Six months after the start of treatment, we rated the left breast cancer as clinical complete response (cCR) and the right breast cancer as clinical stable disease (cSD) evaluated by RECIST (Therasse et al. 2000) (Figure 3). Bilateral modified radical mastectomies with axillary lymph node dissection were performed. The pathological responses were assessed in surgical specimens of breast with reference to the standards of the Japanese Breast Cancer Society (Kurosumi et al. 2008) (Figure 4). In the left surgical specimen included lymph nodes, no cancer cells were observed, thus the therapeutic effect was determined as a pathological complete response (pCR). In contrast, there were residual invasive cancer cells in the right side with two of 12 lymph node metastasis. As adjuvant therapy, she had received trastuzumab mono-therapy for nine months and simultaneously received letrozole for five years. She passed away 71 months after the start of treatment with no recurrence.

## Discussion

In this report, we experienced the patient who presented with synchronous locally advanced bilateral breast cancers with different tumor biology: the left breast cancer was hormone receptor (HR)-negative and HER2-positive, meanwhile the right breast cancer was HR-positive and HER2-negative status. We had planned NAC targeting to the left breast cancer which seemed more progressive disease, thus we administered FEC followed by wPac + T and as expected, those mass showed different mass reduction responses. Importantly, it is recently reported that the HR and HER2 status, breast cancer subtype, are involved with the mass reduction effect of NAC and pathological response. Furthermore, in term of patient’s prognosis, there is different significance of pCR depending on breast cancer subtype (Toi et al. 2008).

It is well known that HER2 overexpression has been associated with benefit from anthracycline-based chemotherapy (Gennari et al. 2008) and trastuzumab-containing chemotherapy, which is a monoclonal antibody targeting the extracellular domain of the HER2 protein (Hudis 2007). As regarding NAC, recent randomized trials present that anthracycline and trastuzumab containing chemotherapy could achieve higher pathological response rates (Gianni et al. 2010;Buzdar et al. 2007). Moreover, treatment response, especially pCR, has a possible surrogate marker of patients’ prognosis after NAC (Von Minckwitz et al. 2012). Taken together, in HER2 type breast cancer, the patients with pCR has a better prognosis than those of non-pCR. Supporting this evidence, even in our locally advanced case, the left tumor resulted in pCR and she has no recurrence for about six years after the start of the treatment.

In related to HR status and systemic therapy, accumulating evidence suggests that the effect of conventional chemotherapy is greater among HR-negative breast cancers (Berry et al. 2006). This is particular striking in the neoadjuvant setting; HR-negative breast cancer is more likely to achieve a pCR to NAC compared to those with HR-positive breast cancer (Ring et al. 2004). HR status is a strong predictive factor of pCR. However, it is really important that pathological response to NAC might not have prognostic significance in patients with HR-positive breast cancer (Von Minckwitz et al. 2012). It must be noted, no mass reduction doesn’t lead to no treatment effect, regarding to prevention of recurrence (Peto et al. 2012). Also in our case, the right mass showed little reduction with a lot of lymph node metastasis remaining. However, fortunately, she has no recurrence for five years after the completion of NAC, though it was more likely to have a recurrence even such a locally advanced and multiple lymph node metastasis.

These days it is common view that breast cancer treatment strategy based on the breast cancer subtypes, considering target therapy to tumor biology as predictive factor for breast cancer oncologist (Goldhirsch et al. 2011). However, at that time, it was largely unknown the meanings of pCR after NAC, and the value of surrogate marker of patients’ prognosis. This bilateral breast cancer case showed typical courses in current treatment strategy through the NAC based on the tumor biology, and a long time follow up showed the meaning of pathological response to NAC based on tumor subtype, even in locally advanced case. This could be the informative experience to understand the relation of treatment response and tumor biology and patient’s prognosis for the various oncology subspecialists.
